# Meningoencephalitis in relapsing polychondritis: A case report

**DOI:** 10.1097/MD.0000000000026315

**Published:** 2021-06-18

**Authors:** Haruki Matsumoto, Ryo Tokimura, Yuya Fujita, Naoki Matsuoka, Tomoyuki Asano, Shuzo Sato, Jumpei Temmoku, Makiko Yashiro-Furuya, Kenji Yoshida, Ryoma Takahashi, Shoko Tanaka, Yuya Itagaki, Mari Honma, Nozomu Matsuda, Hiroshi Watanabe, Kiyoshi Migita, Kazuaki Kanai

**Affiliations:** a Department of Rheumatology; b Department of Neurology, Fukushima Medical University School of Medicine, Fukushima; c Department of Neurology, Masu Memorial Hospital, Nihonmatsu, Fukushima, Japan.

**Keywords:** anti-type II collagen antibodies, cerebrospinal fluid, interleukin-6, meningoencephalitis, relapsing polychondritis

## Abstract

**Rationale:**

Aseptic meningoencephalitis is a rare central nervous system complication of relapsing polychondritis (RP).

**Patient:**

We report a 61-year-old Japanese male patient with spiking fever and impaired consciousness. Neurological examination revealed meningealirritation, and cerebrospinal fluid (CSF) examination showed lymphocytic pleocytosis with elevated protein (199 mg/dL) and interleukin-6 (3810 pg/mL). Serological analysis showed high levels of anti-type II collagen antibodies, and the result of auricular biopsy was consistent with the diagnosis of RP showing cartilage degeneration surrounded by inflammatory cell infiltrations.

**Diagnosis:**

A clinical diagnosis of RP was made according to the diagnostic criteria established by MacAdams et al.

**Intervention:**

Steroid pulse therapy (methylprednisolone 1000 mg, consecutive 3 days) followed by oral prednisolone (60 mg/day) resolved the patient's high fever and disturbance of consciousness.

**Outcomes:**

The patient rapidly improved after steroid treatments and has a normal quality of life under the maintenance dose of steroid plus methotrexate (4 mg/week).

**Lessons:**

RP-associated meningoencephalitis is a rare complication with significant morbidity and mortality. It should be considered and differentiated in patients with RP with unexplained spiking fever and impaired consciousness. In addition, the assessment of cerebrospinal fluid interleukin-6 levels may be useful to investigate the disease activity of RP-related meningoencephalitis. Further prospective studies are required to confirm this result.

## 1 Introduction

Relapsing polychondritis is a rare autoimmune disease characterized by progressive inflammation and destruction of cartilaginous tissues in the ear, nose, and tracheobronchial trees.^[1]^ Although the etiology is unknown, type II collagen is considered a potential autoantigen.^[2]^ This disease has a wide array of clinical manifestations that can mimic rheumatic diseases. However, the involvement of the central nervous system (CNS) is rare with diagnostic dilemma.^[3]^ Here we report a patient with impaired consciousness and cerebrospinal fluid (CSF) pleocytosis with suspicion of infectious meningoencephalitis. However, further exploration led to a diagnosis of RP-related meningoencephalitis.

## 2 Case presentation

A 61-year-old Japanese male patient was referred to our hospital for fever, headache, and confusion. One month before admission, he presented with newly onset fever and headache. A lumber puncture was performed at that time, and CSF showed a raised protein concentration, pleocytosis, and hypoglycorrhachia. Cranial computed tomography (CT) and magnetic resonance imaging (MRI) showed no abnormal findings except for a right parietal lobe lesion due to past head trauma. Initially, infectious meningoencephalitis was suspected, and acyclovir, vancomycin, meropenem, and dexamethasone were administered. Confusional state improved temporarily, and CSF cell count decreased. However, the patient relapsed with delirium and spiking fever. In addition, posterior pain in the left ear appeared. He was transferred to our hospital for further examination.

**Figure 1:**
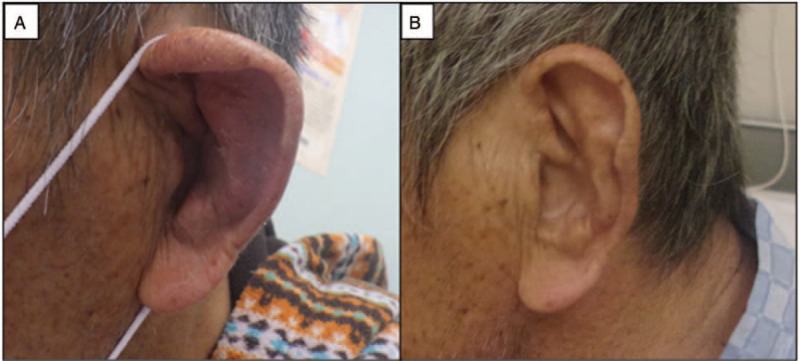
Changes of ear symptom before and after treatment. (A) Floppy-eared appearance of left ear. (B) After glucocorticoid therapy, redness and swollen of left ear was obviously improved.

On admission, the patient's body temperature was 36.7°C, and his blood pressure was 142/88 mmHg. During hospitalization, he had spiking fevers of ≥38°C. A physical examination revealed meningeal irritation and floppy appearance of his left ear (Fig. 1A). He scored 27/30 on the Mini-Mental State Examination, but he then developed recurrent episodes of altered awareness. Occasionally, he was unable to communicate accurately with the medical staff. A neurological examination showed general exaggeration of bilateral deep tendon reflexes and myoclonus of right lower extremity.

Laboratory data showed an inflammatory reaction (Table 1). The patient had an elevated C-reactive protein (CRP) level of 2.09 mg/dL and an elevated erythrocytes sedimentation rate of 70 mm/hr. Antibody to type II collagen was positive with high titers (44.5 EU/mL, <25 EU/mL). The CSF contained 189 cells/μL (mononucleocyte 52.6%, polymorphonuclear cell 47.4%), with a protein level of 199 mg/dL and a glucose level of 36 mg/dL. The oligoclonal bands were negative, but the immunoglobulin G (IgG) index in the CSF was elevated to 1.02. Cultures and polymerase chain reaction testing of the CSF for bacteria, mycobacterium, and herpes simplex viruses were negative. However, the CSF levels of interleukin-6 (IL-6) were significantly elevated (3810 pg/mL). Immunological tests and microbiological tests ruled out other diseases (Table 1). Electroencephalography showed poorly organized posterior dominant rhythm of 8 to 9 Hz and no epileptiform discharges.

**Table 1:** Laboratory findings on admission.

Peripheral blood			
Red blood cells	342 × 10^4^ /μL	IgM	65 mg/dL (50–269)
Hemoglobin	11.7 g/dL	Complement 3	103 mg/dL (73–138)
Hematocrit	34.0%	Complement 4	29 mg/dL (11–31)
Plt	40.3 × 10^4^ /μL	ANA	<160 (0–159)
White blood cells	7,100 /μL	Anti-ds-DNA Ab	(−) (<9.9)
Neutrophil	73%	Anti-SSA Ab	<0.5 U/mL (−) (<6.9)
Eosinophil	1%	Anti-SSB Ab	(−) (<6.9)
Monocyte	9%	PR3-ANCA	(−) (<2.0 U/mL)
Lymphocyte	16%	MPO-ANCA	(−) (<3.5 U/mL)
Baso	1%	Type II collagen Ab	44.5 EU/mL (<25)
**Blood chemistry**		Human Leukocyte Antigen	A2, A11, B60, B61
Total protein	7.1 g/dL	**Microbiologocal tests**	
Total bilirubin	0.7 mg/dL	HBs Ag	(−)
Albumin	3.5 g/dL	Anti-HCV Ab	(−)
Aspartate aminotransferase	18 IU/L (13–30)	HIV-Ab	(−)
Alanine aminotransferase	45 IU/L (10–42)	CMV antigenemia C10C11	(−)
Lactate dehydrogenase	154 IU/L (124–222)	β-D glucan	<6.0 (0–11.0)
Alkaline phosphatase	451 IU/L (106–322)	Tuberculosis specific interferon γ	(−)
Creatine Kinase	22 IU/L (41–153)	Blood culture	(−)
Blood urea nitrogen	20 mg/dL	**Cerebrospinal fluid tests**	
Cr	0.76 mg/dL	Cell count	189/μL (<5)
Ferritin	569 ng/mL	Lymphocyte	52.6%
Na	133 mEq/L	Polymorphonuclear cell	47.4%
K	4.1 mEq/L	Protein	199 mg/dL (10–40)
Cl	94 mEq/L	Glucose	36 m/dL (50–75)
**Blood sugar related tests**		Chloride	115 mmol/L (120–125)
postprandial plasma glucose	91 mg/dL	Oligoclonal band	(−)
**Immunoserological tests**		IgG index	1.02 (0–0.73)
C-reactive protein	2.09 mg/dL (<0.30)	IL-6	3810 pg/mL
Erythrocyte sedimentation rate	70 mm/h (<15)	Cerebrospinal fluid culture	(−)
sIL-2R	506 U/mL (121–613)	HSV PCR	(−)
IgG	857 mg/dL (861–1747)	TB PCR	(−)
IgA	326 mg/dL (93–393)	**Urinalysis**	Normal

**Figure 2:**
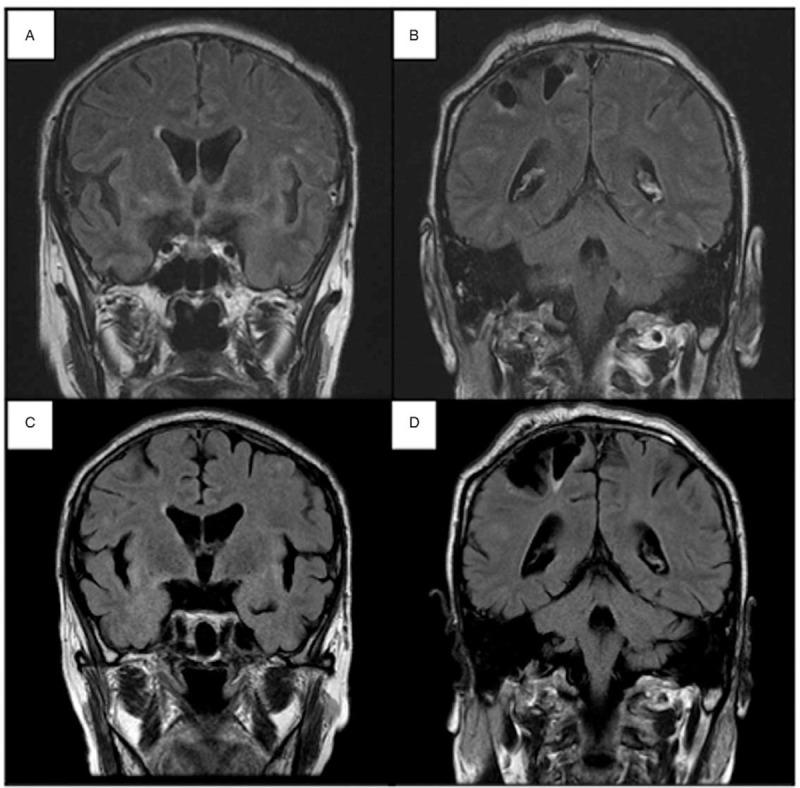
Fluid-attenuated inversion recovery images of brain magnetic response imaging. (A, B) Diffuse hyperintense signals in cerebral sulci and both auricles. (C, D) After treatment, the high intensity signals disappeared. The lesion in the right parietal lobe is due to past trauma.

**Figure 3:**
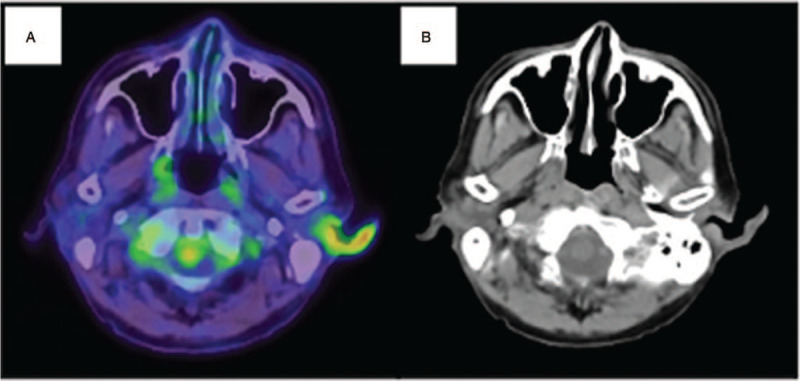
Finding of 18F-FDG positron emission tomography-computed tomography (PET-CT). (A) 18F-FDG PET-CT revealed strong accumulation in both auricles. (B) Metabolic activity lesion was corresponded to mild skin thickening on the CT.

**Figure 4:**
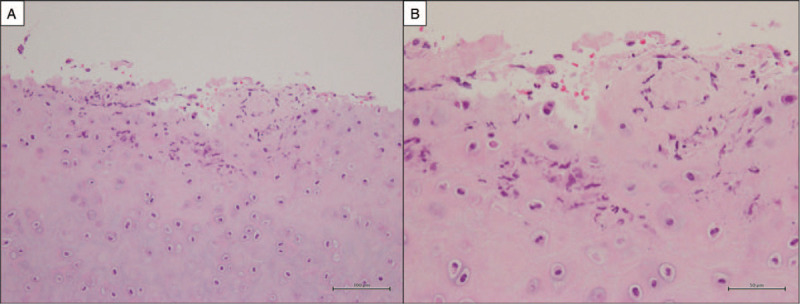
Histological findings of biopsy specimen from the left auricle. Inflammatory cell infiltration of neutrophils around the auricular cartilage was observed (Hematoxylin and Eosin staining, (A) original magnification ×200, (B) original magnification ×400).

Cranial MRI was performed again and fluid-attenuated inversion recovery (FLAIR) images showed diffuse hyperintense signals in cerebral sulci and both auricles (Fig. 2A and B). ^18^F-FDG positron emission tomography-computed tomography revealed strong accumulation in left auricle (Fig. 3). An ear cartilage biopsy showed moderate infiltration of inflammatory cells (neutrophils) around cartilage tissue (Fig. 4). He was diagnosed with relapsing polychondritis (RP) according to McAdam's criteria.^[4]^

The patient was treated with methylprednisolone (mPSL) pulse therapy (1000 mg/d for 3 consecutive days), followed by oral prednisolone (PSL) at a dose of 60 mg/d for 2 weeks. Consequently, his symptoms improved, and oral PSL was gradually tapered. Improvement in cognitive function, other neurological symptoms, and swelling of the auricle were achieved (Fig. 1 B). The high-intensity signals in cerebral surface in the FLAIR images disappeared after the treatment with glucocorticoid therapy (Fig. 2 C and D). The CSF levels of interleukin-6 (IL-6) decreased to 48.8 pg/mL. Methotrexate (MTX) 4 mg per week was introduced when PSL was reduced to 35 mg/day. The patient was discharged, and no relapse occurred at approximately 2 months after treatment.

## 3 Discussion

Relapsing polychondritis is a rare autoimmune disease characterized by systemic inflammation of cartilaginous structures leading to progressive articular or organ damage.^[1]^ Central nervous system involvement is rare, and only 3% of patients with RP develop neurological involvement related to meningeal inflammation or vasculitis in the CNS.^[5–7]^ We presented a 61-year-old male who presented with RP-related symptoms as well as spiking fever and impaired consciousness. CSF analysis showed pleocytosis and elevated protein levels. All viral, bacterial, and paraneoplastic studies were negative. During the clinical course, the patient developed recurrent episodes of altered awareness. MRI showed diffuse FLAIR hyperintense signal throughout cerebral sulci.

This patient presented with subacute encephalopathy mimicking encephalitis or CNS vasculitis. Furthermore, there was no history or findings suggestive of tumor, recent vaccination, or drug or immune-mediated aseptic meningitis. After ruling out these possibilities, the presence of anti-type II collagen antibody led to the diagnosis of RP-related meningoencephalitis.

The etiology of CNS complications in patients with RP remains unknown because of its rarity. Although vasculitis is assumed to be a cause of CNS involvements in RP,^[8]^ brain autopsies performed in patients with RP showed neuronal loss and gliosis with lymphocytic infiltration, suggesting an autoimmune mechanism in its pathogenesis.^[9]^ The patient in this case showed marked pleocytosis with elevated polymorphonuclear cells and markedly elevated levels of IL-6 and IgG in the CSF. CSF analysis in patients with RP with CNS involvement shows abnormalities including pleocytosis and reduced glucose levels that mimic pyogenic meningitis.^[10,11]^ Therefore, we suspected an autoimmune mechanism in the pathophysiological processes of meningoencephalitis seen in this case.

IL-6 is a critical cytokine in the Th17 pathway of T cell development in addition to B cell differentiation or antibody production.^[12]^ Previous reports have suggested that IL-6 is involved in the autoimmune encephalitis, because these patients have elevated levels of IL-6 in the CSF.^[13]^ In contrast to these reports, IL-6 is presumed to be purely injurious to the nervous system.^[14]^ The expressions of the autoantigens, such as collagens, initiate inflammatory responses of RP. Presumably, the deposition of these antigens in the basement membrane of the leptomeninges and parenchymal cerebrovasculature provides sites for antibody-antigen reactions that activate immune cell-mediated attacks on vascular elements.^[15]^ Although a histological analysis was not performed in the present case, it is possible that the deposition of anti-type II collagen antibody in the basement membrane of parenchymal cerebrovasculature provides sites for autoantibody-antigen immune reactions to activate inflammatory processes of vascular elements. Alternatively, activated T cells infiltrate into the CNS to induce inflammatory response in the brain in an antigen-independent manner.

Given the autoimmunity in the pathogenesis of RP, a number of biologics targeting the cytokine-mediated cascades are being used in patients with RP.^[16]^ Kawai et al have reported satisfactory effects of the anti-interleukin-6 receptor antibody tocilizumab in 2 patients with refractory RP.^[17]^ The positive therapeutic role for IL-6 blockage in these reported cases suggests that the elevated IL-6 levels in the affected sites and IL-6 inhibition may therefore disrupt cellular and humoral immune pathway contributing to PR and its organ involvement.

We present a case of RP with high fever and decreased consciousness, which can be halted and completely reversed with immunosuppressive treatments. More evidence is required to assess the efficacy of these agents and to identify the role of IL-6 in the immunopathological processes of RP including CNS involvement. Moreover, early recognition of CNS involvement, in addition to cartilage or articular features of RP, is vital to the timely management of RP, thus allowing for the prevention of irreversible brain damage. Given the immunological mechanisms involved in RP, meningoencephalitis lesions in brain may occur. The present case highlights the importance of CSF IL-6 levels in the screening RP-related meningoencephalitis. There are no known optional therapeutic approaches to RP with meningoencephalitis because of its rarity. Usually, steroid therapy including steroid pulse therapy is administered.^[18]^ Infectiveness or flare-ups may occur with steroid monotherapy, and therefore, the combination of immunosuppressive agents such as azathioprine, MTX, or cyclophosphamide is needed during the course of steroid therapy.^[19]^ In this case, clinical improvement was achieved after combined immunosuppressive treatment consisting of steroid plus MTX.

## 4 Conclusions

Meningoencephalitis is a rare manifestation of RP. Here, we report a patient with RP with meningoencephalitis with severe brain damage and elevated levels of IL-6 in the CSF. The patient recovered completely with steroid therapy. Early diagnosis and prompt therapeutic intervention are important in RP patients with meningoencephalitis. More cases and research are required to identify the pathological role of IL-6 in this rare neurological involvement of RP.

## Acknowledgments

The authors are grateful to Ms Sachiyo Kanno for her technical assistance in this study.

## Author contributions

**Conceptualization:** Haruki Matsumoto, Ryo Tokimura, Yuya Fujita, Naoki Matsuoka, Tomoyuki Asano, Shuzo Sato, Jumpei Temmoku, Makiko Yashiro-Furuya, Kenji Yoshida, Ryoma Takahashi, Shoko Tanaka, Yuya Itagaki, Mari Honma, Nozomu Matsuda, Hiroshi Watanabe, Kazuaki Kanai.

**Supervision:** Kazuaki Kanai, Kiyoshi Migita.

**Writing – original draft:** Nozomu Matsuda, Haruki Matsumoto, Kiyoshi Migita, Kazuaki Kanai.

**Writing – review & editing:** Haruki Matsumoto, Kiyoshi Migita.
